# Prediction of dysnatremias in critically ill patients based on the law of conservation of mass. Comparison of existing formulae

**DOI:** 10.1371/journal.pone.0207603

**Published:** 2018-11-26

**Authors:** Anastasia Katsiampoura, Dimitrios Toumpanakis, Konstantina Konsta, Andreas Varkaris, Theodoros Vassilakopoulos

**Affiliations:** 1 Department of Critical Care, Pulmonary Unit, Evangelismos Hospital, National and Kapodistrian University of Athens, Medical School, Athens, Greece; 2 Anesthesiology Department, St. Elizabeth’s Medical Center, Tufts Medical School, Boston, MA, United States of America; 3 Department of Internal Medicine, Lahey Clinic, Tufts Medical School, Boston, MA, United States of America; 4 Department of Therapeutics, Alexandra Hospital, National and Kapodistrian University of Athens Medical School, Athens, Greece; Azienda Ospedaliero Universitaria Careggi, ITALY

## Abstract

**Background:**

We aimed to examine the predictive value of a novel mathematical formula based on the law of conservation of mass in calculating sodium changes in intensive care unit patients and compare its performance with previously published formulae.

**Methods:**

178 patients were enrolled from 01/2010 to 10/2013. Plasma and urine were collected in two consecutive 8-hour intervals and the sodium was measured. The predicted sodium concentration was calculated based on previous equations and our formula. The two 8-hour period (epoch 1 and 2) results were compared. Variability of predicted values among the measured range of serum sodium levels were provided by Bland-Altman plots with bias and precision statistics. Comparison of the results was performed with the statistical model of the Percentage Similarity.

**Results:**

47.19% patients had dysnatremias. The bias ± SD with 95% limits of agreement for sodium levels were -1.395±3.491 for epoch 1 and -1.623 ±11.1 for epoch 2 period. Bland-Altman analysis for the epoch 1 study period had the following results: -0.8079±3.447 for Adrogué–Madias, 0.56±9.687 for Barsoum–Levine, 0.1412±3.824 for EFWC and 0.294±4.789 for Kurtz–Nguyen formula. The mean similarity, SD and coefficient variation for the methods compared with the measured sodium are: 99.56%, 3.873, 3.89% epoch 1, 99.56%, 1.255, 1.26% for epoch 2, 99.77%, 1.245, 1.26% for Adrogue-Madias, 100.1%, 1.337, 1.34% for Barsoum-Levine, 100.1%, 1.704, 1.7% for Nguyen, 100.1%, 1.370, 1.37% for ECFW formula.

**Conclusions:**

The law of conservation of mass can be successfully applied for the prediction of sodium changes in critically ill patients.

## Introduction

Dysnatremias (hyponatremia and hypernatremia) are common electrolyte disorders in critically ill patients.[1–3] Hypernatremia is defined as serum sodium >145 mmol/L, whereas hyponatremia is defined as serum sodium < 135mmol/L. The symptoms and signs of sodium disturbance are related to the balance between sodium, potassium and water balance. [4] The manifestation of clinical symptoms depends more on the rate of the change in plasma sodium concentration than on the absolute concentration. In chronic hyponatremia, sodium levels drop gradually over 48hours or longer and symptoms are typically more moderate. In contrary, acute hyponatremia causes rapid shifting of water from the extracellular to the intracellular compartment and may result in potentially life-threatening complications including cerebral edema and neurological dysfunction.

Dysnatremias have been associated with prolonged hospitalization and increased intra-hospital mortality.[5, 6] Specifically, approximately 20–30% of patients are diagnosed with dysnatremias at ICU admission, whereas up to 75% of ICU patients develop sodium disturbances. The mortality rate of patients with ICU-acquired dysnatremias (IAD) approaches 30% and 45% for mild and severe sodium disturbance respectively, compared to 16% of patients with normal serum sodium levels. [6, 7] The ICU population is specifically at risk of developing dysnatremia due to pathophysiological derangements or iatrogenic interventions. Inadequate fluid management directly promotes IAD, whereas attempts to correct sodium disturbances based on empirical calculations predispose to high fluctuations in serum sodium concentration that indirectly increase patients’ morbidity and mortality rates.

Numerous formulae have been proposed to predict changes in serum sodium levels including water deficit, (1) sodium deficit, (2)[8] Adrogue-Madias,(3) [9] Nguyen-Kurtz,(4) [10] Barsoum-Levine (5) [11] and Electrolyte Free Water Clearance (EFWC) Eq (6)[12]. The above formulae are weight-based. It should be acknowledged that non-weight-based formulae have also been used for special situations. Accordingly, Bhaskar et al. examined the role of a non-weight based hypertonic saline protocol for the treatment of acute symptomatic severe hyponatremia (serum sodium < 120 meq/L in euvolemic patients with promising results. [13]

These equations have been useful to some extent in clinical practice, however, several limitations associated with the nature of the methods have been identified, including (1) changes in equation variants with therapy (e.g. changes of Total Body Water (TBW)), (2) water and sodium input and output throughout therapy (i.e. high sodium concentration if therapeutic agents), (3) indolent loses of water and sodium.

The majority of these equations were never tested in patients but only on hypothetical cases. Few studies have evaluated these equations in actual patients. The group of Elisaf evaluated the Adrogue-Madias equation but in patients in general medical wards and not in the ICU setting [14]. The only study conducted in ICU patients was by Lindner and colleagues who restricted their analysis to patients with hypernatremia [15]. Although less frequent than hypernatremia, hyponatremia is a significant clinical problem in ICU patients. The first aim of our study was to test and compare the performance of the various proposed equations to predict sodium changes in ICU patients with both hypernatremias and hyponatremias. The second aim of our study was to test a mathematical formula we derived based on the law of conservation of mass and on information readily available at the bedside in the patient’s chart that could accurately predict the changes of sodium concentration in the human body during critical illness and could be easily applied in clinical practice. Although the time interval may be important for the establishment and treatment of serum sodium disturbances, to our knowledge there have been no prospective studies to determine the appropriate time interval for the accurate prediction of sodium changes and a previous study in ICU patients used a 24-hour prediction time interval, which is rather too long for the ICU patients [15]. We instead used an 8-hour prediction time interval and hypothesized that we could predict the sodium concentration the ensuing 8 hours based on data readily available to the physician designing the dysnatremia correction strategy. The sodium prediction model was applied prospectively during 2 consecutive 8-hour periods (epoch 1 and epoch 2, to account for the changing patient status in the ICU and its effect on the prediction) and both models were compared to the measured sodium concentration at the end of the respective 8-hour period. The new equation was compared with the above-mentioned previously proposed formulas to predict sodium change.

## Materials and methods

### Study population

From January 2010 to October 2013, we enrolled 178 adult patients that were hospitalized in the multidisciplinary (trauma surgical & medical) Intensive Care Unit of Evangelismos General Hospital of Athens, Greece. Patients of all ages, gender, underlying diseases, anesthesia and mechanical ventilation status were included in the study. The exclusion criteria for this study were impossible arterial blood drawing, patients on continuous renal replacement therapy and absence of a urine catheter. General patients’ characteristics are shown in S1 Table.

### Study design

**Fig 1:**
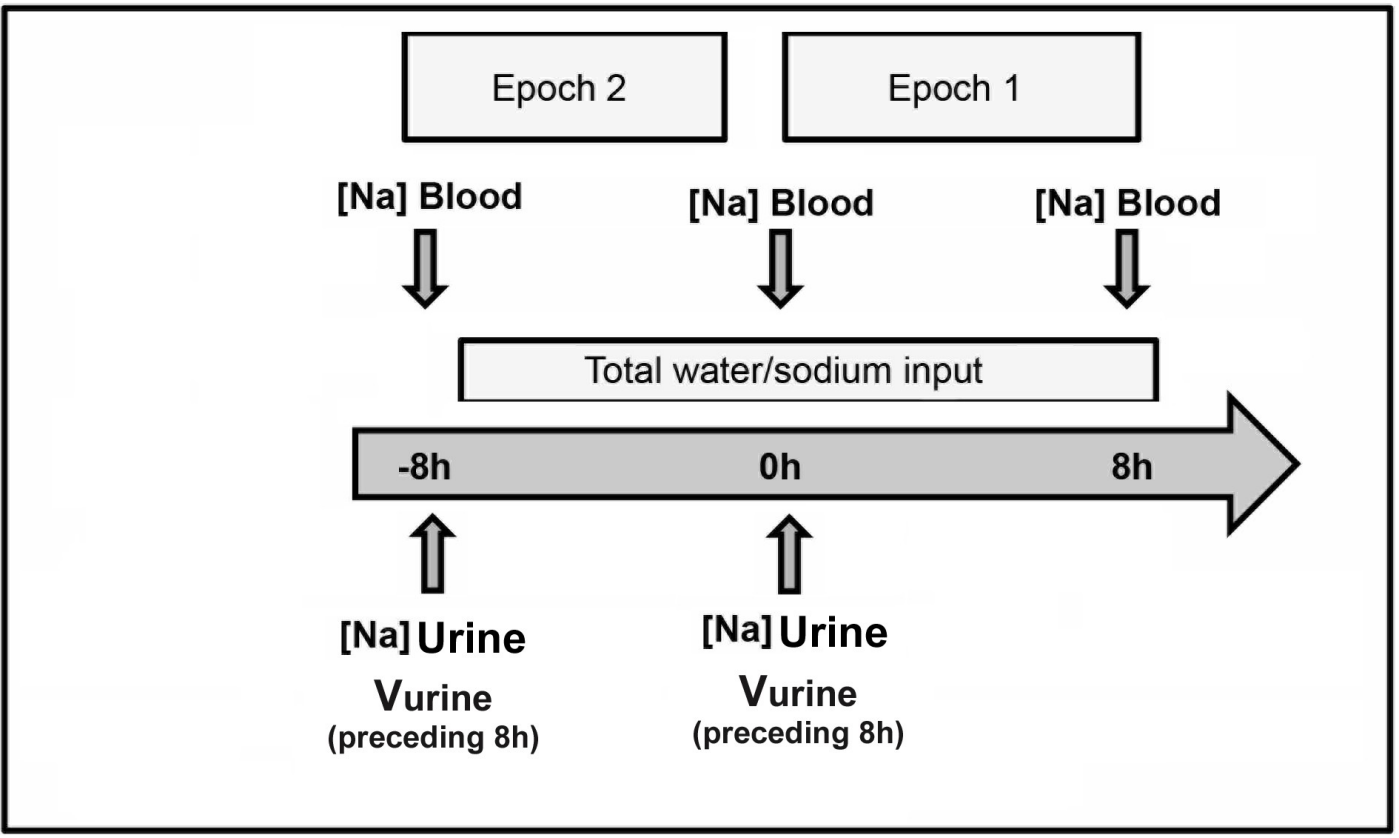
Representative schema of study period.

We divided this study in two 8-hour intervals, epoch 1 and epoch 2 (Fig 1). Arterial blood samples were collected at baseline (t = 0h, initial) and the end of each epoch respectively (t = +8h, final). The Na,_urine_ was measured at t = 0h of each epoch. The urine volume was measured from the preceding 8 hours of each epoch (t = 0 to t = -8h) based on the patient’s chart and was assumed to be close to the urine volume during the respective epoch. The arterial blood was collected either by an arterial line or by arterial catheterization. Blood (4ml) was obtained from radial artery and urine (5ml) was obtained with a syringe from the urine catheter. Both blood and urine samples were analyzed by an automated computerized gas analyzer (Ciba Corning 238 pH/Blood Gas Analyzer; Ciba Corning Diagnostics Ltd, Halstead, UK). During the two intervals, total water and sodium inputs and outputs were recorded (S2 Table), including drug-related input/output, enteral and parenteral solutions. The sodium concentration and volume of each solution was provided by the pharmacy. We did not exclude from the study patients receiving insulin treatment. Patient’s weight was measured at the time of each blood draw. Patients were measured in their beds using a bed scale without disconnecting lines or monitoring equipment at any time during the measuring procedure. Verbal or written informed consent was obtained from each patient or patient’s representative before the inclusion in the study. Evangelismos General Hospital of Athens Institutional Review Board and Ethics Comity approved the protocol of the study.

### Mathematical formula

Prediction of plasma sodium concentration changes was obtained based on a novel mathematical formula:

$$\begin{array}{l} {\left[Na^{+}\right]}_{initial}\times \mathrm{TBW}+{\left[Na^{+}\right]}_{infused}\times V_{infused}+{\left[Na^{+}\right]}_{drug}\\ \;\times V_{drug}+{\left[Na^{+}\right]}_{enteral}\;x\;V_{enteral}\\ \;+{\left[Na^{+}\right]}_{parenteral}\;x\;V_{parenteral}-{\left[Na^{+}\right]}_{urine}\times V_{urine}\\ \;={\left[Na^{+}\right]}_{final}\times \left(\mathrm{TBW}\pm \mathrm{equilibrium}\right) \end{array}$$ (1)

Where TBW is Total Body Water.

We calculated the Total Body Water (TBW) as actual body weight (kg) x 50% for both genders and all patient ages. Actual body weight was measured for every patient on the day of the study. The “equilibrium” of our formula is the difference in volume between total inputs and outputs.

Finally, we used previously introduced formulae for the prediction of sodium changes in our population, including the Adrogue-Madias, the Barsoum-Levine, the Kurtz-Nguyen and the EFWC formula. These formulae were designed for the prediction of the serum sodium concentration after the infusion of 1L of solution. For the comparison of the predictive power of our equation with other existing formulas we used the following formulae that were mathematically derived by Lindner et al [12]:

Adrogue–Madias formula:

$${Na}_{2}=\frac{{(Na}_{1}\times TBW\times )+[{Vol}_{inf}\times {\left(Na+K\right)}_{inf}}{TBW+{Vol}_{inf}}$$ (2)

Barsoum–Levine formula:

$${Na}_{2}=\frac{(TBW+{Na}_{1})+[{Vol}_{input}\times {\left(Na+K\right)}_{input}-{Vol}_{out}\times {\left(Na+K\right)}_{out}}{TBW+\Delta Vol}$$ (3)

EFWC formula:

$${Na}_{2}=\frac{{Na}_{1}\times TBW}{TBW-EFWC}$$ (4)

Kurtz–Nguyen formula:

$${Na}_{2}=\frac{\left[(Na+23.8)\times TBW]+[1.03\times [{\left(Na+K\right)}_{input}-{\left(Na+K\right)}_{out}]\right]}{TBW+\Delta Vol}-23.8$$ (5)

EFWC according to Rose:

$$EFWC=\frac{{Vol}_{urine}\times (1-({{Na}^{+}+K^{+})}_{urine}}{{Na}_{serum}}$$ (6)

The sodium correction strategy was decided by the treating physician who was blind to the results of the predictive models tested in this study.

### Statistical analysis

Predicted changes were compared with actual changes in sodium concentration as measured by arterial blood sampling. The variability of predicted values among the measured range of serum sodium levels were provided by Bland-Altman plots with bias and precision statistics.

Comparison of the results of the different equations was performed with the statistical model of the Percentage Similarity. Percentage Similarity histograms were compared to the measured sodium (gold standard) simultaneously. We calculated the coefficient of variation for further definement of the agreement between the results of the different equations on our population.[16]

## Results

We analyzed a total of 178 individual cases. In our cohort, eighty-four (47.19%) patients had dysnatremias at the time of their inclusion in the study, 17 (9.55%) had hyponatremia and 67 (37.64%) had hypernatremia. The mean plasma [Na^+^] at baseline was 143.5 mEq/L (range 129–164 mEq/L). 135 (75.8%) patients were receiving enteral solutions during the recording period, whereas 108 (60.7%) where treated with parenteral solutions. The mean urine [Na^+^] was 99.2 mEq/L (range 11–214 mEq/L). Changes in plasma [Na^+^] as measured by the gold standard method were observed in 142 patients (80%). Seventy-one patients (40%) had changes in plasma [Na^+^] >2meq and 39 patients (22%) had changes >5meq.

**Fig 2:**
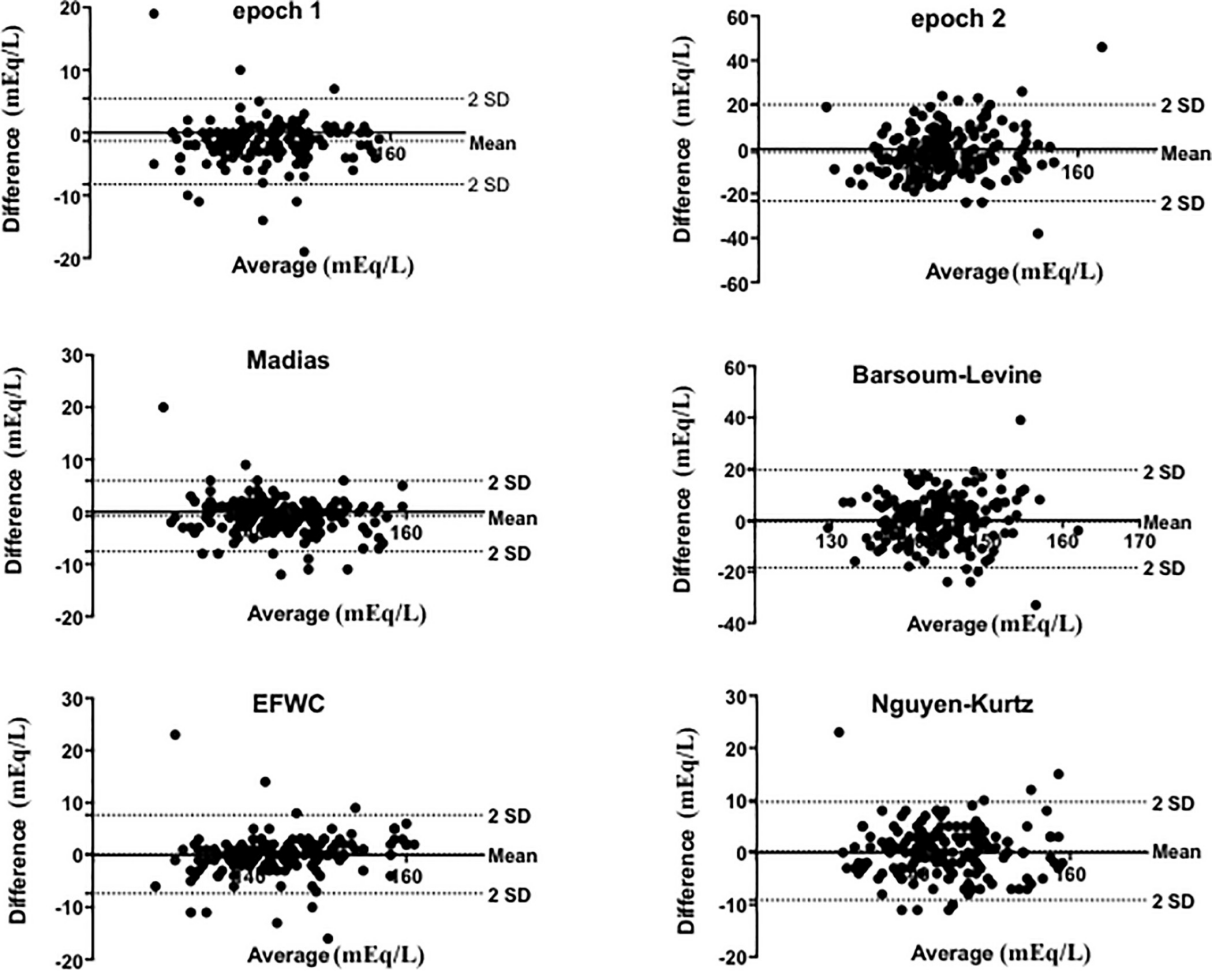
Bland & Altman diagram of the sodium concentration values in our population. A) epoch 1 period, B) epoch 2 period, C) Madias equation, D) Barsoum-Levine, E) EFWC equation, and F) Nguyen-Kurtz on our population.

A scatter diagram of the sodium concentration values measured in our population and predicted by our formula is shown in Fig 2. Data are presented graphically using a Bland-Altman plot. The bias ± SD with 95% limits of agreement for sodium levels were -1.395±3.491 (-8.327 to 5.446) for our epoch 2 and -1.623 ±11.1 (-23.39 to 20.14) for our epoch 1 study period. Bland and Altman analysis applied to the other previously described equations for the prediction of sodium changes in our population for the epoch 2 study period had the following results: -0.8079±3.447 (-7.564 to 5.949) for the Adrogué–Madias formula, 0.56±9.687 (-18.43 to 19.55) for the Barsoum–Levine formula, 0.1412±3.824 (-7.345 to 7.363) for the EFWC formula and 0.294±4.789 (-9.093 to 9.681) for the Kurtz–Nguyen formula.

**Fig 3:**
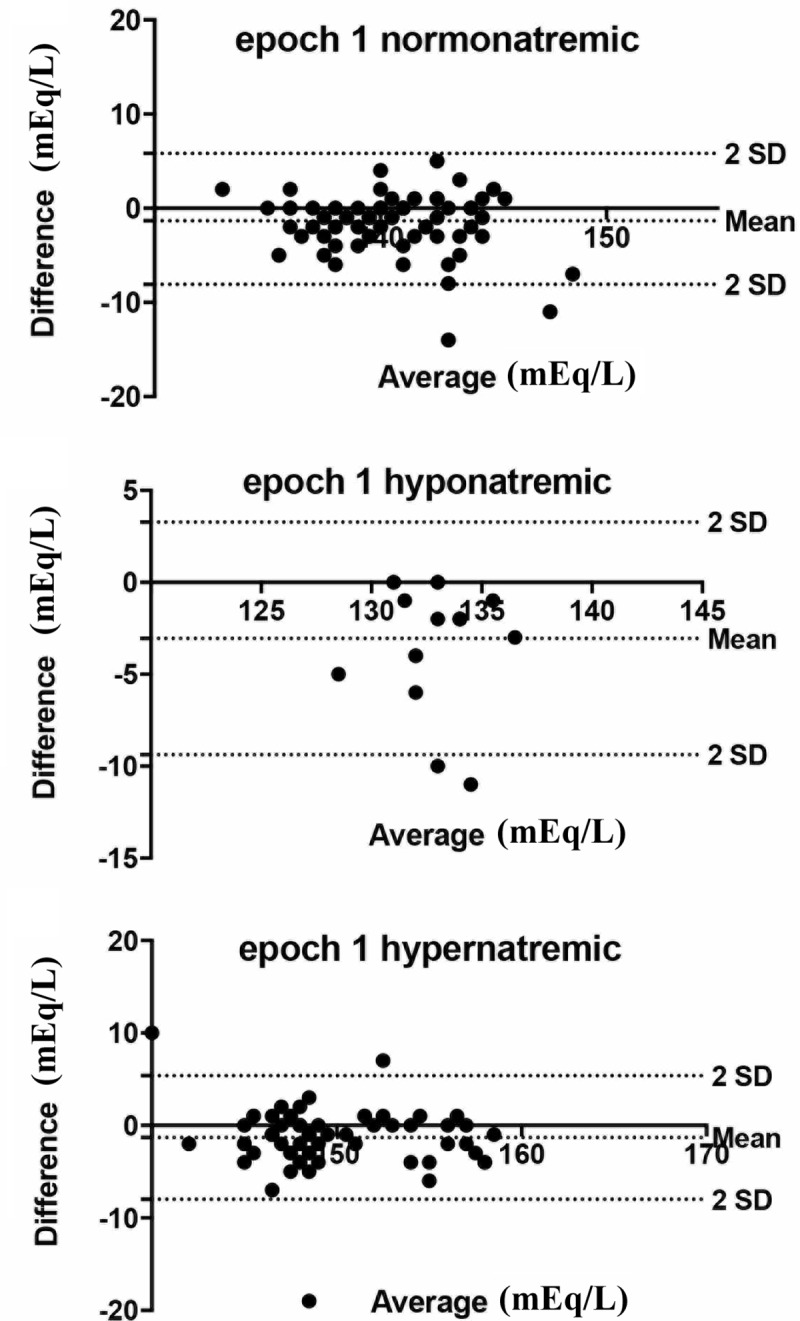
Bland & Altman diagram of sodium concentration values in patient subpopulations. A) hyponatremic, B) normonatremic, and C) hypernatremic patients for the epoch 1 study period.

Subgroup analysis for hyponatremic and hypernatremic patients on their enrollment was performed for our equation. In hyponatraemic patients bias± SD with 95% limits of agreement were: -3.056±3.226 (-9.397 to 3.268) in hypernatremic patients: -1.304±3.410(-7.988 to 5.379) while in normonatremic: -1.133±3.458 (-8.088 to 5.821) (Fig 3).

**Fig 4:**
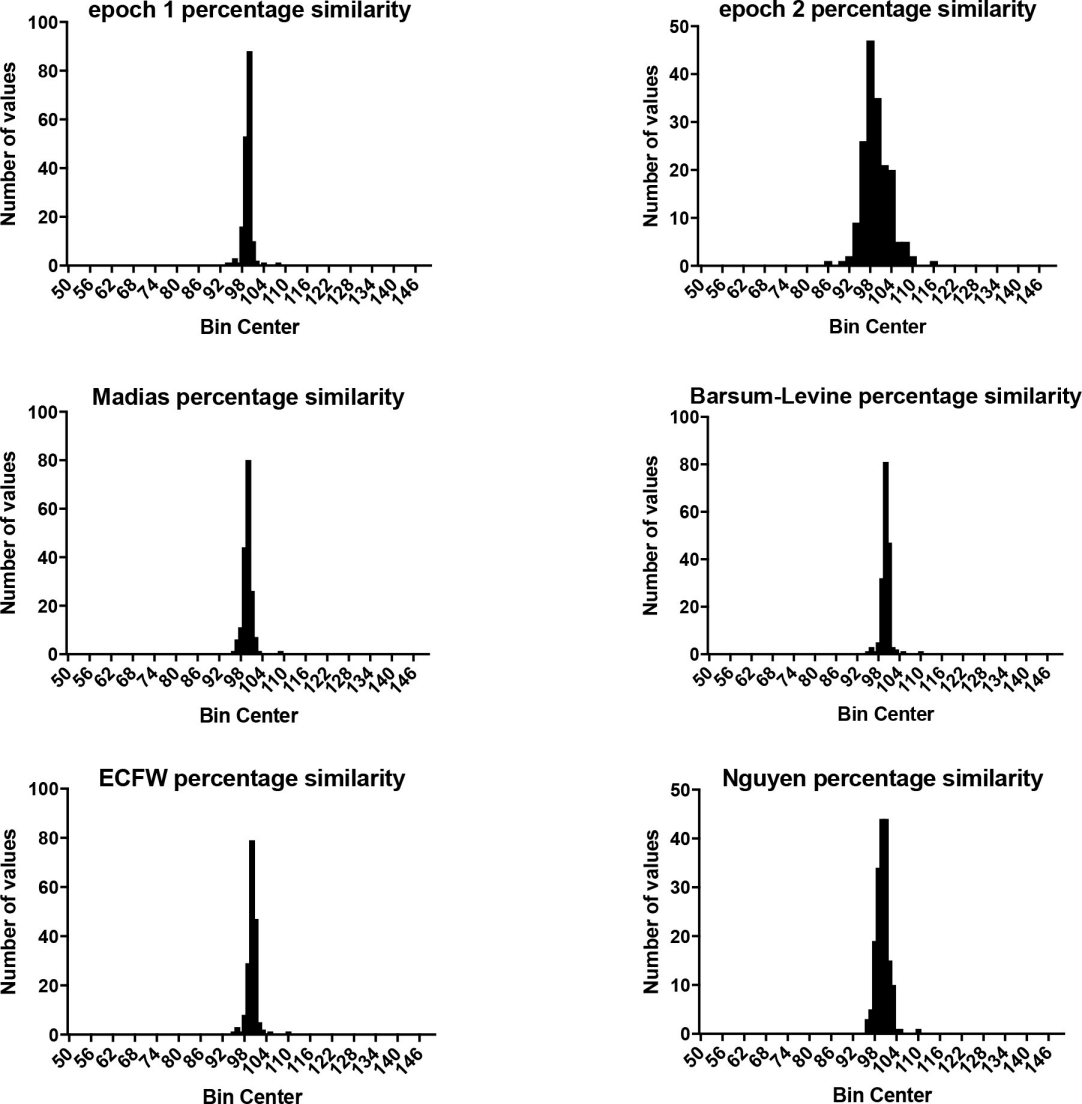
Comparison of the predictive power of our equation with other existing formulae with the statistical model of the percentage similarity. A) epoch 1, B) epoch 2 period, C) Madias equation, D) Barsoum-Levine equation, E) EFWC equation, and F) Nguyen-Kurtz on our population.

Comparison of the predictive power of our equation with other existing formulae was performed using the statistical model of the Percentage Similarity. The mean similarity, SD and coefficient variation for the methods compared with the measured sodium are: 99.56%, 1.255, 1.26% for our equation applied on the epoch 2 study period, 99.56%, 3.873, 3.89% for the epoch 1 study period, 99.77%, 1.245, 1.26% for the Adrogue-Madias equation, 100.1%, 1.337, 1.34% for Barsoum-Levine equation, 100.1%, 1.704, 1.7% for the Nguyen equation, 100.1%, 1.370, 1.37% for the ECFW equation (Table 1 and Fig 4).

**Table 1:** Comparison of the predictive power of our equation with other existing formulae with the statistical model of the percentage similarity.

	Mean Similarity	Standard Deviation of similarity (SD)	Mean Percentage Difference ± SD	Coefficient of Variation
**Madias**	99.77%	1.245%	-0.23±1.245%	1.26%
**Barsoum-Levine**	100.1%	1.337%	+0.10±1.337%	1.34%
**Nguyen**	100.1%	1.704%	+0.10±1.704%	1.70%
**ECFW**	100.1%	1.370%	+0.10±1.370%	1.37%
**Epoch 1**	99.53%	1.255%	-0.47±1.255%	1.26%
**Epoch 2**	99.56%	3.873%	-0.44±3,873%	3.89%

## Discussion

Based on our findings, the law of conservation of mass can be applied for the accurate prediction of sodium changes in critically ill patients. In our population dysnatremias were encountered in 71% of the patients included in the study. These derangements have been associated with numerous untoward clinical effects and increased mortality in ICU patients, primarily due to disastrous empirical treatment attempts. Thus, it is critical to be able to (1) predict changes in sodium concentration following iatrogenic interventions and (2) estimate the volume and type of infusion needed to properly correct dysnatremias.

To address this challenging clinical problem, numerous mathematical formulae have been proposed, and clinically tested. Edelman et al.[17] were the first to propose the correlation between serum sodium concentration and serum osmolarity. They assumed that total inputs and outputs of sodium and water determine the changes in serum sodium. Based on the Edelman hypothesis[17], two simple formulae (1) Sodium deficit formula and (2) Water deficit formula were proposed to estimate sodium and water static deficits respectively. [18] However, a potential limitation of these formulae is that they do not evaluate the effect of the administered fluids on the serum sodium concentration. Adrogue-Madias [9] proposed a formula that was designed for the prediction of the serum sodium concentration after the infusion of 1 liter of any solution. An omission of this this formula is that it approaches the human organism as a closed system and thus (1) it does not take into account concurrent water and electrolyte losses from the kidneys (through urine) that affect the sodium concentration, (2) assumes that TBW is unchanged, (3) the gastrointestinal and skin losses are not evaluated. Another limitation of this formula is that it does not take into consideration the contribution of the non-active osmotically sodium molecules that are stored in the third space to the change of the sodium concentration a correlation that Edelman had proposed [14]. In an attempt to overcome the Adrogue-Madias formula’s limitations, Barsoum–Levine equation included the renal losses into calculations. However, this equation does not include additional inputs of electrolytes or water through enteral and/or parenteral sources that affect the sodium balance. Furthermore, TBW changes or the gastrointestinal and skin losses were not eval uated. Clinical evaluation showed that Barsoum and Levine’s formula “overcorrects hyponatremia”.[12] Recently Linder et al proposed the EFWC formula [12]. This formula is based on the renal clearance mechanism that was first described by Rose [8]. Specifically, these authors suggested that the kidneys are the major water balance regulator, and thus they are the major contributors to sodium concentration balance. This formula accepts the dynamic nature of TBW but it does not take into consideration total infused water and sodium. A clinical evaluation of the formula showed that it is not valid for the prediction of sodium changes when great water and sodium losses are present. Finally, based on Edelman’s studies, Kurtz-Nyugen hypothesized that sodium concentration should be calculated not in the plasma, but in the plasma water which is equal to the 93% of the whole plasma [10]. Moreover, with this formula TBW changes according to the input and output of fluids and sodium. This formula applies for the prediction of sodium disturbances in SIADH and hyperglycemias.

Unlike the majority of previous studies that evaluated the proposed formulae with one or a few hypothetical clinical examples we evaluated our formula as well as previously published formulae with a prospective clinical study of 178 real critically ill patients. Only Linder et al evaluated their predictive equation along with other equations in 66 normonatremic and hypernatremic but not hyponatremic patients. However, these investigators did not apply any statistical analysis to actually compare the various predictive equations, but only Bland and Altman analysis to compare the individual equations to the gold standard which is the actual measurement of sodium. To our knowledge our study is the clinical study evaluating all available predictive formulae in the largest cohort of critically ill patients and comparing them using appropriate statistical methodology. No other head to head comparison of the existing sodium prediction formulae in critically ill patients using appropriate statistics is available.

In this prospective study we examined if a simple mathematical formula, using total water/sodium inputs and outputs during 8hour intervals could predict changes in plasma sodium concentration. We assumed that our equation, based on the law of conservation of mass, could predict the sodium concentration in the critically ill patients. We included the total sodium and water inputs not only by the infused fluids but also by the parenteral and the enteral fluids. Total outputs were calculated as urine volume and urine sodium. Our formula is based on the TBW changes for the prediction of sodium concentration by the calculation of the equilibrium (ΔVolume), which reflects the changes in the TBW according to the total fluid inputs and outputs during the study period. We did not exclude from our population patients with hyperglycemia or SIADH.

According to the subgroup analysis our formula works effectively for the prediction of sodium changes with less bias in the normonatremic and hyponatremic population in comparison with the hypernatremic population of our study. We investigated the predictive performance of our equation using data collected in two consecutive 8-hour periods (epochs 1 & 2). This was done on the premise that the critically ill patient’ s clinical status changes continuously, so our equation might perform differently during the varying clinical status in the two different epochs. Our study demonstrates that the predictive performance of our equation was slightly better in epoch 2 than in epoch 1, though these differences were not clinically significant.

Our study has limitations. We did not include indolent sodium losses due to the difficulty to estimate these losses in the clinical setting, despite the assumption that these losses affect water-electrolyte balance and might affect the precision of our study. We have chosen not to include potassium measurement in our newly proposed predictive formula for a variety of reasons. First for simplicity and cost containment. In addition, as elegantly discussed by Ring T in the original work of Edelman et al, wherein potassium was included, the patients were not studied in steady-state conditions but under sodium restriction, whereas exchangeable sodium, exchangeable potassium and total body water (TBW) were not measured simultaneously. [19] The statistical approach used by Edelman and colleagues to derive their proposed equation is challenging. For the entire data set, they obtained linear regression equations by the method of least squares and found the data were described by the equation [Na] = 1.11*(Nae+Ke)/TBW-25.6. The use of simple regression analysis for the cross-sectional data containing one data point per individual to infer the relationship between x and y within any individual raises important statistical concerns, especially in the absence of replicate data within the same individual. The original report of Edelman et al, both the intercept and the slope of the proposed equation were given without confidence intervals. However, Edelman et al. reported a standard deviation from regression of 5.6, from which can be computed a standard deviation of 0.069 for the slope, and hence by a t-test on 96 degrees of freedom 1.11 is not significantly different from 1. Similarly, a standard deviation for the intercept is 10.3, yielding a 95% confidence interval of -5.2 to -46.0. Hence, the intercept is largely undefined in accordance with the fact that the minimal measured value on the abscissa is 125.3. Importantly, no account was taken in the regression for the fact that the X value ((Nae+Ke)/TBW) was measured with error. However, since the study reported errors of measurement for each component of the quotient, it is easy to verify by sampling from the normal distribution that this further significantly attenuates the slope and increases the intercept by making the relationship to [Na] flatter. Thus, it is not certain at all that the relationship is linear (fitting a spline model does indicate a curvature) and certainly the slope is not determined with absolute certainty; in particular, the intercept is ill defined, i.e. the degree of certainty regarding the magnitude of this constant term is not great. [20]Thus, we cannot take as given that the situation of each set of patients matches that set studied by Edelman et al. [19] Of note, the correlation between sodium concentration and total exchangeable potassium is poor. Finally, and most important, correlation does not prove causality. In fact, Edelman et al did not actively change the concentration of potassium in patients, so as to measure the effects of this change on the measurement of sodium. Thus, the equation they obtained is purely correlative. Despite omitting potassium, our predictive equation was at least performing equally well with those predictive formulae incorporating potassium. Another potential limitation of our study is the measurement of patient’s weight. Measurement of patient’s actual weight is a major challenge is daily clinical practice, specifically in critically ill patients that are traditionally weighted in their beds. In our study, we used a bed scale which to our knowledge is the method of preference in ICU practice. (REF: Determination of body weight and height measurement for critically ill patients admitted to the intensive care unit: A quality improvement project. [21]

The TBW was calculated as estimation according to the body weight and was the same for both genders and all ages, although this compromises accuracy. Another limitation of the study is the consideration of steadily secreted sodium and urine volume during the 8h interval, a hypothesis that is not completely valid for patients with acute kidney injury. We have used an automated computerized gas analyzer for the measurement of sodium and not values obtained by the laboratory. Although laboratory values may be more accurate, we have chosen to use the gas analyzer values because we wanted to be as clinically practical as possible, using sodium values at the point of care, where the managing physician is prescribing orders without introducing the delay of sending the sample to the laboratory and waiting for the results. We excluded patients on renal replacement therapy from the current study and this another limitation of our study. Finally, our study is a single center study, and external validation in other ICU populations is required before our results could be generalized.

In this study we demonstrate that using total sodium/water input and output in the prospective study period can reliably predict changes in sodium concentrations. Based on our results, we suggest that changes sodium/water balance during the prospective study period determine changes in serum sodium concentration. Although mathematical formulas have rather limited use in the ICU daily practice, correction of dysnatremias is an exception. Our formula could apply for the treatment of sodium disorders of critically ill patients in everyday clinical practice, since it allows an accurate replacement of fluid losses in order to target the required serum sodium levels. The amount of the solution needed to correct the serum sodium level can be calculated by the following formula:

$$\begin{array}{l} V_{\mathrm{infused}}=\frac{{\left[{\mathrm{Na}}^{+}\right]}_{\mathrm{final}}\times \left(\mathrm{TBW}\pm \mathrm{equilibrium}\right)}{{\mathrm{Na}}^{+}{}_{\mathrm{infused}}}\\ \;-\frac{{\left[{\mathrm{Na}}^{+}\right]}_{\mathrm{initial}}\times \mathrm{TBW}-{\left[{\mathrm{Na}}^{+}\right]}_{\mathrm{drug}}\times V_{\mathrm{drug}}-{\left[{\mathrm{Na}}^{+}\right]}_{\mathrm{enteral}}\times V_{\mathrm{enteral}}}{{\mathrm{Na}}^{+}{}_{\mathrm{infused}}}\\ \;-\frac{{\left[{\mathrm{Na}}^{+}\right]}_{\mathrm{parenteral}}\;{x\;V}_{\mathrm{parenteral}}\;{\left[{\mathrm{Na}}^{+}\right]}_{\mathrm{urine}}\times V_{\mathrm{urine}}}{{\mathrm{Na}}^{+}{}_{\mathrm{infused}}} \end{array}$$ (7)

We believe that our formula is a step forward in achieving better (i.e more precise) correction of sodium disturbances. Since our formula is effective for the prediction of dysnatremias in critically ill patients, we speculate that this formula could be predictive of the sodium disturbances in other patient populations. This hypothesis needs to be tested in large-scaled prospective studies.

## Supporting information

S1 Table Patient characteristics. (DOCX)

S2 Table Total inputs and outputs in the study population. (DOCX)
