# Supplementary material for: Prediction of dysnatremias in critically ill patients based on the law of conservation of mass. Comparison of existing formulae
Source: PLoS One. 2018 Nov 26;13(11):e0207603. doi: 10.1371/journal.pone.0207603 (PMC6261024; doi:10.1371/journal.pone.0207603)
Supplement: S1 Table — (DOCX) [file pone.0207603.s001.docx]

**Supplementary Table 1: Patient characteristics**

| **Patient characteristics** | | | |
| --- | --- | --- | --- |
| **Sex** | **Number** | **Percentage** |  |
| **Male** | 128 | 72% |  |
| **Female** | 50 | 28% |  |
|  |  |  |  |
|  | **Mean** | **Range** | **SD** |
| **Age (y)** | 72 | 23-89 | 18.3 |
| **Weight (kg)** | 90 | 60-180 | 20.1 |
| **Baseline [Na]** | 144 | 128-173 | 7 |
