# Supplementary material for: Prediction of dysnatremias in critically ill patients based on the law of conservation of mass. Comparison of existing formulae
Source: PLoS One. 2018 Nov 26;13(11):e0207603. doi: 10.1371/journal.pone.0207603 (PMC6261024; doi:10.1371/journal.pone.0207603)
Supplement: S2 Table — (DOCX) [file pone.0207603.s002.docx]

**Supplementary Table 2: Total inputs and outputs in the study population**

| Total Inputs | Total Outputs |
| --- | --- |
| Intravenous fluid | Urine (-8h): Volume and sodium concentration |
| Solvent solutions for medications (sodium content of antibiotics was included) | Urine (0h): Volume and sodium concentration |
| Enteral solutions |  |
| Parenteral solutions |  |
